# Gene Transfection of Human Turbinate Mesenchymal Stromal Cells Derived from Human Inferior Turbinate Tissues

**DOI:** 10.1155/2016/4735264

**Published:** 2015-12-13

**Authors:** Jin Seon Kwon, Seung Hun Park, Ji Hye Baek, Truong Minh Dung, Sung Won Kim, Byoung Hyun Min, Jae Ho Kim, Moon Suk Kim

**Affiliations:** ^1^Department of Molecular Science and Technology, Ajou University, Suwon 443-749, Republic of Korea; ^2^Department of Otolaryngology-Head and Neck Surgery, The Catholic University of Korea, College of Medicine, Seoul 137-701, Republic of Korea

## Abstract

Human turbinate mesenchymal stromal cells (hTMSCs) are novel stem cells derived from nasal inferior turbinate tissues. They are easy to isolate from the donated tissue after turbinectomy or conchotomy. In this study, we applied hTMSCs to a nonviral gene delivery system using polyethyleneimine (PEI) as a gene carrier; furthermore, the cytotoxicity and transfection efficiency of hTMSCs were evaluated to confirm their potential as resources in gene therapy. DNA-PEI nanoparticles (NPs) were generated by adding the PEI solution to DNA and were characterized by a gel electrophoresis and by measuring particle size and surface charge of NPs. The hTMSCs were treated with DNA-PEI NPs for 4 h, and toxicity of NPs to hTMSCs and gene transfection efficiency were monitored using MTT assay, fluorescence images, and flow cytometry after 24 h and 48 h. At a high negative-to-positive charge ratio, DNA-PEI NPs treatment led to cytotoxicity of hTMSCs, but the transfection efficiency of DNA was increased due to the electrostatic effect between the NPs and the membranes of hTMSCs. Importantly, the results of this research verified that PEI could deliver DNA into hTMSCs with high efficiency, suggesting that hTMSCs could be considered as untapped resources for applications in gene therapy.

## 1. Introduction

Stem cells could be categorized into two main types: embryonic stem cells (ESCs) that are derived from the inner cell mass of blastocysts [1] and adult stem cells (ASCs) [2], which are separated from a variety of adult tissues of mammals [3]. ESCs show pluripotency and the ability to differentiate into the endoderm, mesoderm, and ectoderm, but are associated with ethical issues because the embryo must be destroyed in the process of cell harvesting [4, 5]. On the other hand, ASCs can be isolated from adult tissues without any ethical problems and show the self-renewal or differentiation characteristics into other types of stem cells existing in the same germ layer [6, 7]. One suggested disadvantage of ASCs is the limitation of the types of stem cells into which they can differentiate. However, this issue has also been overcome following recent studies demonstrating the potential of stem cell transdifferentiation to extraneous cells with their origin tissues [8–10]. The most commonly used ASCs for regenerative medicine are mesenchymal stem cells (MSCs), especially MSCs derived from bone marrow (BMSCs), because of their characteristics such as ease of isolation and fast proliferation* in vitro*. Furthermore, other types of MSCs have been discovered and separated from various adult tissues such as fat, umbilical cord blood, dental tissues, placenta, and peripheral blood [11–15], for use in the fields of tissue engineering and regenerative medicine.

Human turbinate-derived mesenchymal stromal cells (hTMSCs) are regarded as a type of MSCs that are isolated from the eliminated inferior turbinate tissues in nose. The procedure for obtaining bone marrow for harvesting hBMSCs is associated with a high level of pain, and therefore new approaches are needed. However, hTMSCs can be separated from the tissues discarded following a turbinectomy or septoplasty owing to hypertrophy of the nasal inferior turbinate tissues. The MSC-like characteristics of hTMSCs have been confirmed using CD markers in previous studies [16]. In addition, hTMSCs can proliferate rapidly* in vitro*, similar to other types of MSCs, and show the ability to differentiate into osteoblast- and chondrocyte-like cells* in vitro* [16–18], as well as bone-like tissues in a hydrogel system* in vivo* [19]. According to these characteristics of hTMSCs, they can also be considered as promising cell sources for tissue engineering and regenerative medicine.

Gene therapy is an attempt used to heal illnesses at the level of DNA, with the potential to cure chronic granulomatous disease, immunodeficiency, cancer, and other complicated diseases [20–22]. Gene therapy involves the intracellular introduction of foreign genes using a virus or nonviral system containing a specific site for physical or chemical attachment to DNA. The information encoded in DNA is transferred to mRNA via transcription; thereafter, the mRNA combines with tRNA and prepares the chain of amino acids for formation of the protein. This is a major advantage of gene therapy, in that specific cells are created with a desired function by controlling protein synthesis using DNA. Moreover, this gene-based therapy could target specific diseases [23]. Thus, desirable and tailorable stem cells can be prepared using gene transfer, and stem cells can be applied for the regeneration of various tissues using a gene delivery system.

Polyethyleneimine (PEI) is most well-known polymeric gene transfer carrier in the field of nonviral gene delivery [24, 25]. It has amine groups in every repeating unit in its molecular structure, which allows it to combine with DNA and form complexes [26]. Furthermore, the protonated amines of PEI in the endosomes promote the inflow of ions and destruction of the endosome membrane. This process could be a possible mechanism for the delivery of DNA into nuclei and the subsequent elimination of PEI [27]. In a previous study, the transfection efficiencies between linear and branched PEI were compared at the same concentration, and the efficiencies were higher when branched PEI was applied [28]. These results verified that the number of primary amines in PEI influences the gene transfection efficiency.

The overall objective of the present research was to transfect enhanced green fluorescence protein (EGFP) genes to hTMSCs using a branched PEI-containing gene carrier. We addressed the following questions in this study. (1) Are hTMSCs appropriate cell sources for gene therapy? (2) Is PEI an effective gene carrier for hTMSCs? (3) Could we apply this gene delivery system for adjusting the expression of specific proteins of hTMSCs in further studies? Resolving these questions would provide evidence of the applicability of hTMSCs in gene therapy and regenerative medicine.

## 2. Materials and Methods

### 2.1. Materials

Branched PEI (10 kDa) and rhodamine B isothiocyanate (Rhod B ITC) were purchased from Sigma-Aldrich (St. Louis, MO, USA), and pEGFP-N2 encoding a red-shifted variant of the wild-type green fluorescence protein was purchased from Clontech (BD Biosciences; Palo Alto, CA, USA).

### 2.2. Isolation and Culture of hTMSCs

Fresh inferior turbinate tissue was obtained from the discarded tissue obtained from a young woman (age 22) who underwent partial turbinectomy at the Catholic University of Korea, St. Mary's Hospital. The protocols of this study were recognized by the Internal Review Board for Human Subjects Research and Ethics Committee (KIRB-00399_18-005), and informed assent was acquired from the patient before enrollment in this experiment. The obtained inferior turbinate tissue was washed 3–5 times using a saline solution with gentamicin (Kukje Pharmaceutical Industries; Sungnam, Korea). The tissue was then washed three more times with Antibiotic-Antimycotic solution (Gibco; Gaithersburg, MD, USA), and twice more with phosphate-buffered saline (PBS), before being cut into pieces (1 mm^3^). The washed tissue was placed in a culture dish in Dulbecco's Modified Eagle's Medium (Gibco) containing 10% fetal bovine serum (FBS; Gibco BRL; Grand Island, NY, USA) and incubated at 37°C in an atmosphere of 5% CO_2_. The medium was changed to fresh medium every 2-3 days. The cells floating on culture plates were separated from the tissue fragments. The obtained hTMSCs were cultured in cell growth medium with 10% FBS and 1% penicillin-streptomycin (Gibco BRL) in minimal essential medium-alpha (MEM-*α*) on a tissue culture flask (BD Falcon; San Jose, CA, USA) at 37°C and under 5% CO_2_. The hTMSCs were stained with CD34 (hematopoietic-positive marker), CD90 (MSCs marker), and CD166 (MSCs marker) antibodies on the surface of cells and were evaluated using flow cytometry (BD Bioscience) for confirming their stemness properties as MSCs. The viabilities of hTMSCs were assured using trypan blue staining before seeding. The gene transfection was performed at 70% confluence of hTMSCs.

### 2.3. Extraction of DNA and Preparation of DNA-PEI Nanoparticles (NPs)

The plasmid DNA was cultivated in* Escherichia coli* (strain DH5*α*) and then endotoxin-free cDNA was extracted using PureLink HiPure Plasmid Filter Midiprep Kit (Invitrogen; Löhne, Germany) following the manufacturer's protocol. The purity and concentration of DNA were measured with a nanodrop spectrophotometer (ND-1000 UV/Vis Spectrophotometer; Thermo Scientific; Wilmington, DE, USA). The DNA was diluted to a 1 mg/mL concentration in Tris-EDTA buffer. The PEI solution dissolved in distilled water (DW) was added to 1 *μ*g of DNA at various negative-to-positive (N/P) charge ratios of 1, 2, 4, 8, 12, and 16, respectively. The solution of DNA-PEI was vortexed immediately and incubated for 30 min to form NPs.

### 2.4. Characterization of DNA-PEI NPs

The formation of DNA-PEI NPs was verified by carrying out gel electrophoresis on 1.2% agarose gels in Tris-acetate-EDTA buffer (AMRESCO; Solon, OH, USA) and visualized using a UV image station (UVP; BioDoc-It Imaging System; Upland, CA, USA) with ethidium bromide (AMRESCO; Solon, OH, USA). The particle size and surface zeta potential of DNA-PEI NPs were measured by dynamic light scattering (ELSZ-1000; Otsuka Electronics; Osaka, Japan) at room temperature.

### 2.5. Cytotoxicity Test

The hTMSCs were sown on 24-well plates (Nunc; Roskilde, Denmark) with 2 × 10^4^ cells/well density and incubated for 24 h before being treated with the DNA-PEI NPs. The cells were washed with serum-free MEM-*α* to remove FBS and replaced in fresh MEM-*α*. The hTMSCs were treated with each of the DNA-PEI NPs for 4 h and then the medium was replaced by cell growth medium. After 24 h and 48 h of transfection with each N/P charge of DNA-PEI NPs, a cytotoxicity test was performed with the 3-(4,5-dimethylthiazol-2-yl)-2,5-diphenyltetrazolium bromide (MTT) assay, a colorimetric assay used to measure the activity of cellular enzymes that decrease the water-soluble MTT reagents to its insoluble purple-colored formazan. In brief, 100 *μ*L of the MTT solution (5 mg/mL in PBS) was added to each well of hTMSCs and the plates were incubated at 37°C in a 5% CO_2_ atmosphere. After 4 h, the entire medium was removed, 500 *μ*L of dimethyl sulfoxide (DMSO) was added, and the plates were incubated for 30 min under 100 rpm shaking to solve the formazan crystals. The optical density at 590 nm was measured using an enzyme-linked immunosorbent assay plate reader (EL808 Ultra Microplate Reader; Bio-Tek Instruments; Winooski, VT, USA). All experiments were accomplished in triplicate.

### 2.6. Transfection Efficiency

The hTMSCs were plated 5 × 10^4^ cells/well density in a 12-well plate (Nunc) at 24 h before transfection. Two micrograms of DNA was added to each well in the form of DNA-PEI NPs with various N/P charge ratios. The hTMSCs were treated with each ratio of DNA-PEI NPs for 4 h. After 24 h and 48 h of transfection, the treated hTMSCs were monitored to confirm the expression of EGFP using Axio Imager A1 (Carl Zeiss Microimaging GmbH; Göttingen, Germany), analyzed with AxioVision Rel. 4.8 software (Carl Zeiss Microimaging GmbH), and harvested through trypsinization to assess transfection efficiency. Harvested hTMSCs were washed with PBS and fixed with 4% paraformaldehyde (Biosesang Inc.; Gyeonggi, Korea) for 24 h. The hTMSCs were resuspended in 100 *μ*L of iced PBS. The green fluorescence of transfected hTMSCs was detected using a FACSCanto II flow cytometer (BD Biosciences) with 520 nm and 570 nm bandpass filters for EGFP. For all samples, 10,000 events were acquired, and data analysis was performed using BD FACSDiva Software (BD Biosciences).

### 2.7. Synthesis of Rhod-PEI

To monitor the insertion time of DNA-PEI NPs into hTMSCs, Rhod B ITC, a red fluorescence dye, was used to label the 5% primary amine groups of PEI. PEI was dissolved in 0.1 M sodium carbonate at 2 mg/mL concentration. Fifty microliters of Rhod B ITC solution in dry DMSO (1 mg/mL) was slowly dropwised to the PEI solution and stirred for 24 h at 4°C. After 24 h, ammonium chloride solution in DW was introduced at a final concentration of 50 mM to quench the reaction. Rhod-PEI was dialyzed using a membrane filter (1000 Da, Spectrum Laboratories Inc.; Rancho Dominguez, CA, USA) for elimination of unreacted Rhod B ITC and diluted in DW at the same concentration with normal PEI.

### 2.8. Acquisition of Time-Dependent Images of Transfected hTMSCs

Real-time images of transfected hTMSCs with DNA-Rhod-PEI were captured and evaluated to assess the insertion time of DNA-Rhod-PEI NPs and intracellular expression of pEGFP. hTMSCs were seeded 3 × 10^4^ cells/well of density on a 2-chamber slide (Thermo Fisher Scientific; Grand Island, NY, USA) and incubated for 24 h. The hTMSCs were treated with 5 *μ*g/mL Hoechst 33342 solution in culture medium in 20 min to stain the nuclei and were then washed with serum-free MEM-*α*. DNA-Rhod-PEI NPs prepared with N/P 8 were added to the hTMSCs. After 4 h, the NPs were removed and the culture medium was added to the transfected hTMSCS. Expression of Rhod-PEI, pEGFP, and Hoechst 33342 in hTMSCs at different time points was monitored using an OLYMPUS IX51 inverted fluorescence microscope (Olympus; Tokyo, Japan, with a Moticam Pro 285A CCD camera) and analyzed with Motic Images Advanced software (Motic; Xiamen, China).

### 2.9. Statistical Analysis

All statistical analyses were conducted through one-way ANOVA analysis of variance with Bonferroni's post hoc test using SPSS 12.0 software (SPSS Inc.; Chicago, IL, USA).

## 3. Results

### 3.1. Characterization of hTMSCs

**Figure 1:**
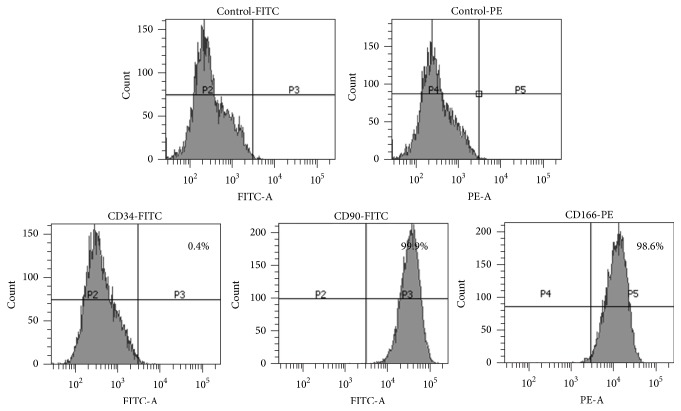
Cell-surface marker expression of hTMSCs using CD34 (negative) and CD90 and CD166 (positive) for confirming their stemness properties as MSCs.

The hTMSCs, derived from the inner tissues of the nose, were easily isolated from the donated tissue after turbinectomy or conchotomy. For characterization of the hTMSCs, flow cytometry was conducted to evaluate the expression of the specific markers at the fifth passage: CD34 (blood cell antibody) as a negative marker and CD90 and CD166 (MSCs-related markers) as positive markers. As a result, the CD34-positive rate of hTMSCs was 0.4%, indicating that hTMSCs are not blood-derived cells. By contrast, over 95% of the cells were CD90- and CD166-positive (99.9% and 98.6%, resp.), indicating that hTMSCs have MSC-like features (Figure 1). Moreover, in accordance with previous research, the hTMSCs rapidly proliferated from passages one to five [18]. These properties of hTMSCs suggest their potential in various fields requiring MSCs and their applicability in gene therapy for curing diseases.

### 3.2. Characterization of DNA-PEI NPs

**Figure 2:**
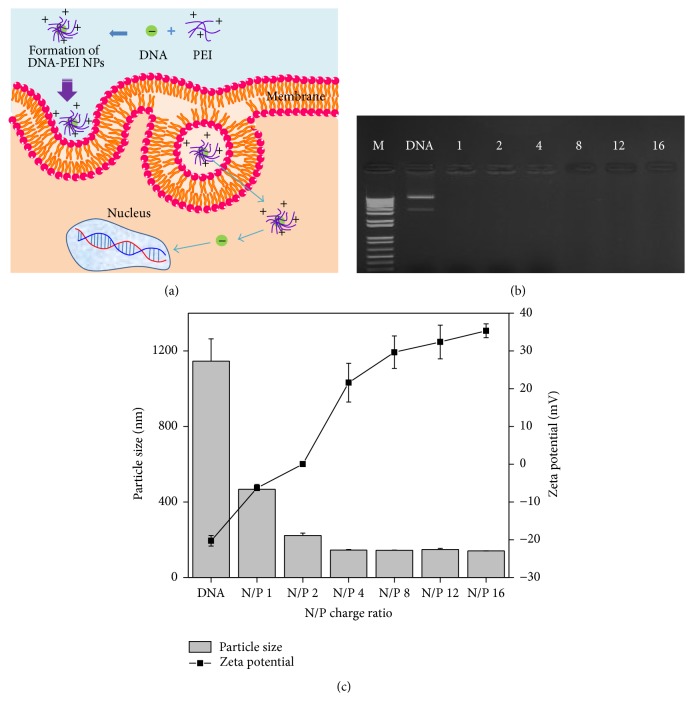
(a) Schematic diagrams of endocytosis using DNA-PEI NPs and the results of (b) electrophoresis on a 1.2% agarose gel and (c) particle sizes and zeta potentials of the DNA-PEI NPs.

The cellular uptake mechanisms of gene transfer using PEI are still unidentified, although endocytosis is the most potent mechanism of nonviral gene transfection using nanosized particles (Figure 2(a)). In this study, DNA-PEI NPs were prepared with various N/P ratios ranging from 1 to 16 to assess complex formation and transfection efficiencies to hTMSCs depending on the N/P charge ratio. The complexation of DNA with PEI was evaluated through an agarose gel retardation assay. The results of electrophoresis showed that the DNA migration was completely retarded by complexation with the positively charged PEI at every N/P ratio (Figure 2(b)). The particle size and zeta potential were measured for characterization of the DNA-PEI NPs. As shown in Figure 2(c), the particle size of DNA was above 1000 nm; however, after addition of PEI, the particle size of the DNA-PEI NPs clearly decreased in a PEI concentration-dependent manner. The zeta potential of DNA was −20 mV because of the phosphate groups in DNA; however, the zeta potentials of the DNA-PEI NPs increased with the addition of more PEI. These results indicate that the DNA-PEI NPs condensed and changed to positively charged particles with the addition of PEI. The nanosized and positively charged particles could enable the insertion of DNA into hTMSCs by endocytosis and attraction.

### 3.3. Cytotoxicity of DNA-PEI NPs to hTMSCs

**Figure 3:**
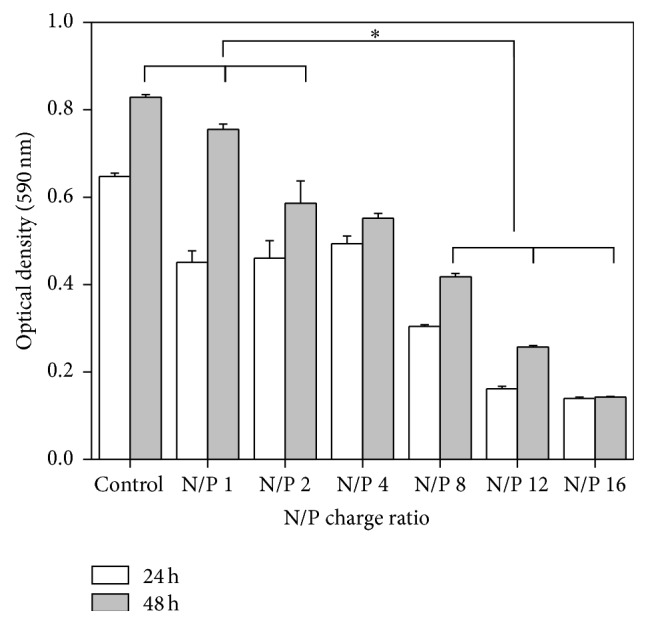
Viability of hTMSCs treated with DNA-PEI NPs at various N/P charge ratios, measured by the MTT assay (^*∗*^ *P* < 0.001).

The cytotoxicity of DNA-PEI NPs to hTMSCs was measured using an MTT assay at a wavelength of 590 nm.  Figure 3 revealed the viabilities of hTMSCs at 24 h and 48 h after treatment with DNA-PEI NPs. The cytotoxicity of DNA-PEI NPs to hTMSCs steadily increased in accordance with increasing N/P charge ratios. From N/P 1 to 8, the hTMSCs showed significant toxicity compared to the control group, but they still proliferated till 48 h after treatment of DNA-PEI NPs. However, the percent viability of hTMSCs was below 50% with treatment of N/P 12 at 24 h and 48 h; at an N/P charge ratio of 16, the optical density of hTMSCs at 48 h was similar to that at 24 h. This result indicated that DNA-PEI NPs with an N/P charge ratio of 16 are the most toxic to hTMSCs and inhibit cell growth.

### 3.4. Transfection Efficiencies to hTMSCs

**Figure 4:**
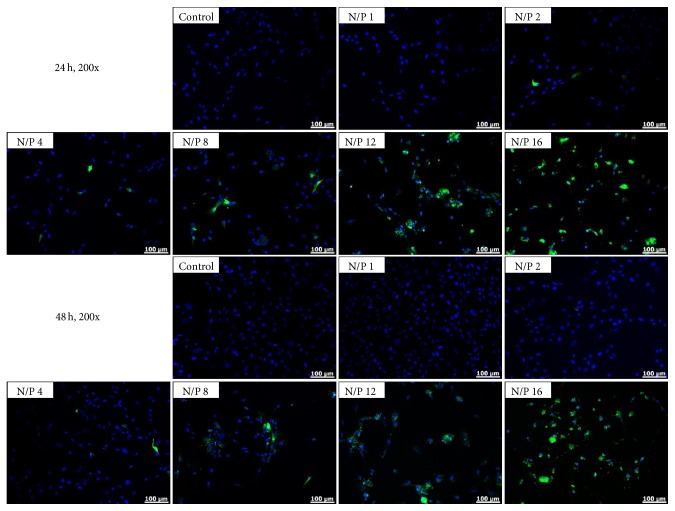
Fluorescence images of hTMSCs after treatment with DNA-PEI NPs. EGFP shows green fluorescence and the DAPI-stained nucleus is indicated as blue fluorescence. Magnification is ×200 and the scale bar represents 100 *μ*m.

**Figure 5:**
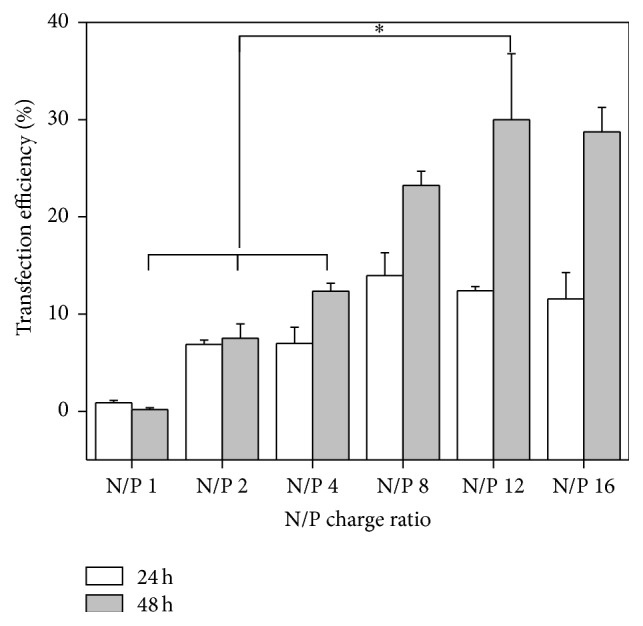
Transfection efficiency of hTMSCs treated with DNA-PEI NPs at N/P charge ratios of 1–16 (^*∗*^ *P* < 0.001).

After introducing the plasmid with EGFP into cells, green fluorescence protein was synthesized in the membrane of the cells, which allowed for the gene transfection efficiency to be evaluated through measuring the expression level of green fluorescence. To monitor transfection efficiencies, the hTMSCs were treated with DNA-PEI NPs at N/P charge ratios of 1–16, and the green fluorescence was detected with a fluorescence microscope (Figure 4) and flow cytometry (Figure 5). Green fluorescence expression was not observed in the nontreated hTMSCs, whereas the green fluorescence of DNA-PEI-treated hTMSCs was enhanced in accordance with the increment of N/P charge ratios. However, at an N/P charge ratio of 16, the transfection efficiency was similar to that at an N/P charge ratio of 12, and it was difficult to directly compare the cells at other N/P ratios. These results indicate that the transfection efficiency was poor with a high amount of PEI and that DNA-PEI NPs with an N/P charge ratio of 16 were too toxic to the hTMSCs, which reduced the number of cells. These results are in accordance with the cell viability analysis.

### 3.5. Time-Dependent Images of Transfected hTMSCs

**Figure 6:**
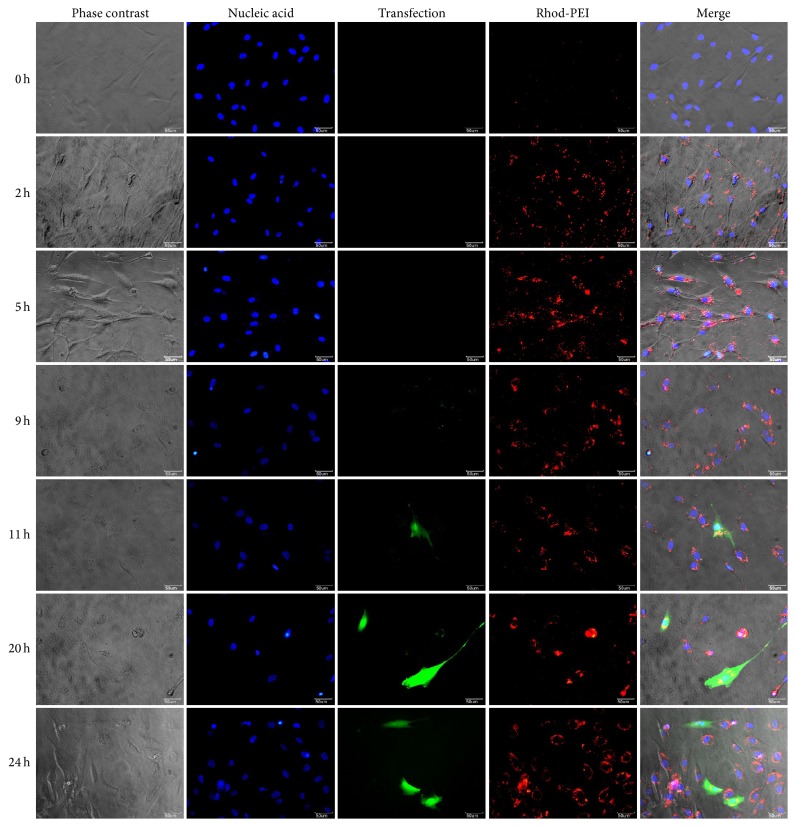
Intracellular expression of EGFP and Rhod-PEI in hTMSCs treated with DNA-PEI NPs at an N/P charge ratio of 8. Time-dependent images of the intracellular expression of EGFP and Rhod-PEI were captured immediately after treating the cells with DNA-PEI NPs. EGFP shows green fluorescence, Hoechst 33342-stained nuclei show blue fluorescence, and Rhod-PEI shows red fluorescence. Magnification is ×400 and the scale bar represents 50 *μ*m.

To assess the insertion of DNA-PEI into the hTMSCs, PEI was labeled with the red fluorescence dye rhodamine B ITC, and DNA-Rhod-PEI NPs were prepared by adding Rhod-PEI solution to DNA with an N/P charge ratio of 8 (Figure 6). Immediately after treatment, we observed phase and blue fluorescence (nuclei) in the images. At 2 h after adding the DNA-Rhod-PEI NPs, there was no fluorescence expression of pEGFP, but the red fluorescence (Rhod-PEI) was observed near the nuclei. This demonstrated that the DNA-Rhod-PEI NPs had attached to the membrane of hTMSCs within 2 h, and the NPs became closer to the nucleic acids of hTMSCs at 5 h. The red fluorescence of Rhod-PEI was weaker at 7 h compared to that at 5 h. The green fluorescence of plasmid DNA was expressed in the nucleic acids of hTMSCs at 9 h, and as of 11 h, distinct green fluorescence was observed in the membrane of hTMSCs. After 24 h, the red fluorescence had slightly moved away from the nucleus of the cells. These results indicated that DNA-PEI was inserted from 2 h to 7 h, and green fluorescence protein was synthesized from nucleic acids at 9 h after treating the cells with DNA-Rhod-PEI NPs. After 24 h, the Rhod-PEI leaked out of the cells and stayed in the cytoplasm of hTMSCs.

## 4. Discussion

The stromal cells used in this study, hTMSCs, were isolated from human inferior turbinate tissue and characterized as MSCs. hTMSCs can differentiate into other types of adult cells such as chondrocytes, adipocytes, and osteoblasts [16–19, 29]. Because of these characteristics, hTMSCs have been considered as critical resources in the treatment of disease in the fields of tissue engineering and regenerative medicine. To assess the feasibility of hTMSCs as promising sources for gene therapy, we conducted gene delivery to hTMSCs and evaluated the transfection efficiency using PEI as a nonviral gene carrier.

PEI has been used as a gene carrier because it contains many amine groups in the main chain of its chemical structure, which are positively charged in solution state, making it possible to transfer a gene into the cells and protect the DNA by the proton sponge effect of PEI [27]. In our work, after adding the PEI solution to DNA, the characteristics of the DNA-PEI NPs were evaluated through a variety of methods such as electrophoresis on an agarose gel and measurements of particle size and zeta potential. The particle size of DNA-PEI NPs decreased from 1145 nm to 140 nm in accordance with increasing PEI concentration due to condensation between DNA and PEI [26], and the zeta potential increased from −20 mV to 30 mV by increasing the positively charged amine groups. DNA-PEI NPs are formed by the electrostatic interaction between DNA and nanosized particles [30, 31].

We evaluated the cytotoxicity and transfection efficiency of hTMSCs after 4 h treatment of DNA-PEI NPs. The cytotoxicity of DNA-PEI NPs with higher N/P charge ratios was higher than that of DNA-PEI NPs with low N/P charge ratios; on the other hand, the green fluorescence of hTMSCs was enhanced by introducing DNA into the cells. PEI likely shows cytotoxicity in gene delivery [32–34] because it increases the intracellular pH by disrupting the pH regulation mechanism and depolarizing the cell membrane [35]. Uptake of NPs is considered to be an adhesion process; therefore, in the case of positively charged NPs, the particles could strongly interact with the cell membrane [36]. As observed in previous studies, the DNA-PEI NPs showed greater cytotoxicity to hTMSCs compared to other MSCs. However, the transfection efficiency of hTMSCs was about twice as high as that observed with other MSCs in previous studies under the same experimental conditions [25, 37]. These results demonstrate that hTMSCs are a great cell source for gene transfection. PEI, the most well-known polymeric gene carrier, could also be used as gene carriers for hTMSCs; however, the cytotoxicity of PEI has to be resolved before effective gene transfection can be achieved.

To monitor the uptake time and intracellular green fluorescence expression of transfected hTMSCs over time, hTMSCs were treated with DNA-Rhod-PEI NPs with an N/P charge ratio of 8. At the initial time point, there was no fluorescence observed, but after a couple of hours, the red fluorescence of Rhod-PEI was detected on the cytoplasm surface of hTMSCs. Green fluorescent protein was expressed in the nucleus of hTMSCs at 9 h after treatment of DNA-Rhod-PEI NPs, which was demonstrated by the colocalization of green fluorescence with the Hoechst stain of nucleic acids. At a longer incubation time, EGFP expression was observed in the cytoplasm and nucleic acids of hTMSCs, and the Rhod-PEI dissociated from the nucleus of hTMSCs. In previous studies, the dissociation of DNA-carrier complexes was observed after uptake into the nucleic acids of cells by DNA polymerase during transcription [38]. However, in our study, the DNA was transfected into hTMSCs, and then the red fluorescence of Rhod-PEI was detected in the cytoplasm of hTMSCs by dissociation of DNA-Rhod-PEI NPs.

Few studies have examined the transfection of specific gene using lentiviral vectors and specific differentiation of stem cells [39]. In this work, although, to the best of our knowledge, we provide the first evidence that DNA-PEI NPs induce gene transfection of hTMSCs, further studies are currently underway to exploit BMP-2 gene delivery into hTMSCs using nonviral gene carrier and to investigate osteogenic differentiation of the transfected hTMSCs.

## 5. Conclusion

We evaluated the introduction of DNA into hTMSCs using a common gene carrier, PEI, to confirm the applicability of hTMSCs in gene therapy. DNA-PEI NPs were formed at various N/P charge ratios by the interaction between DNA and PEI. Greater cytotoxicity of transfected hTMSCs was detected for DNA-PEI NPs with higher N/P charge ratios using the MTT assay. The transfection efficiency of hTMSCs was about 30% using PEI as a gene carrier at N/P 12. Our results demonstrated that PEI, a general nonviral gene carrier, is applicable for gene transfection of hTMSCs and that hTMSCs are a potential resource for gene therapy because their gene transfection efficiencies were higher than those of other MSCs. Through this research, we could verify that hTMSCs are promising cell sources for gene transfection* in vitro*, and this system could be applied for controlling the expression of specific proteins depending on the types of genes transfected. However, the optimized formulation of DNA and PEI that might achieve high transfection efficiency in the low cell toxicity are needed as further studies.
